# Glut9-mediated Urate Uptake Is Responsible for Its Protective Effects on Dopaminergic Neurons in Parkinson’s Disease Models

**DOI:** 10.3389/fnmol.2018.00021

**Published:** 2018-01-26

**Authors:** Mingxia Bi, Qian Jiao, Xixun Du, Hong Jiang

**Affiliations:** Department of Physiology, Shandong Provincial Key Laboratory of Pathogenesis and Prevention of Neurological Disorders and State Key Disciplines: Physiology, Medical College, Qingdao University, Qingdao, China

**Keywords:** Parkinson’s disease, urate, urate oxidase, Glut9, p53, MPTP

## Abstract

Considerable evidence has shown that elevated plasma or cerebrospinal fluid (CSF) urate levels correlated with a reduced risk of Parkinson’s disease (PD). Based on its anti-oxidative properties, urate might serve as one of promising neuroprotective candidates for PD. However, how urate is transported through cell membranes to exert its effects inside the cells in PD is largely unknown. To elucidate this, we showed that increased intracellular urate exerted its neuroprotective effects against 1-methyl-4-phenylpyridinium (MPP^+^)-induced neurotoxicity in MES23.5 cells and elevated urate could antagonize 1-methyl-4-phenyl-1,2,3,6-tetrahydropyridine (MPTP)-induced nigral dopaminergic neuronal death in urate oxidase (UOx) knockout (KO) mice. Its transporter, glucose transporter type 9 (Glut9), was observed up-regulated, which was caused by the activation of p53. These protective effects could be abolished by Glut9 blocker and p53 inhibitor. These results suggested that Glut9 was a functional urate transporter, whose up-regulation by activation of p53 resulted in the increased intracellular urate levels in PD models. Our findings suggest that Glut9 could be modified to modulate urate levels in dopaminergic neurons and urate-elevating strategies without increasing systemic levels to avoid side effects might serve as a potential therapeutic target for PD.

## Introduction

Parkinson’s disease (PD) is a common neurodegenerative disorder characterized by a progressive loss of dopaminergic neurons in the substantia nigra (SN) and subsequent dopamine depletion in the striatum (Hornykiewicz, 1998; Song et al., 2017). The exact etiology and pathogenesis responsible for dopaminergic neuronal degeneration are largely unknown. Accumulating evidence indicates that oxidative stress is a major contributor in the pathogenesis of PD (Jenner and Olanow, 1998; Schapira et al., 1998), suggesting that antioxidants could have some potential usages in PD treatment.

Urate, an end product of purine metabolism in humans, is a natural and powerful antioxidant (Ames et al., 1981; Becker, 1993). Although high plasma urate levels are risk factors for several metabolic syndromes, such as gout and type 2 diabetes mellitus, they could decrease the incidence of PD and its progression (Hayden and Tyagi, 2004; Schwarzschild et al., 2008; Ascherio et al., 2009). Davis et al. (1996) first described men with serum urate levels above the median had a decreased rate of idiopathic PD. Subsequent epidemiological and experimental studies all suggest that higher dietary urate intake or early diagnosis of gout has a lower risk of PD (Alonso et al., 2007; Gao et al., 2008). In addition, reduced urate levels in the SN have been observed prior to clinical symptoms in PD patients (Church and Ward, 1994), which is more likely a predictor of PD diagnosis in the prodromal phase and neuroprotective intervention before the onset of neurological symptoms (Fang et al., 2013; Ascherio and Schwarzschild, 2016).

The neuroprotective effects of urate in PD have been studied by several groups. For example, urate could alleviate the impaired motor performance in PD animal models *in vivo* and prevent the degeneration of dopaminergic neurons *in vitro* (Gong et al., 2012; Zhu et al., 2012). Notably, these effects of urate are shown to depend on its intracellular levels (Cipriani et al., 2012a; Zhang et al., 2014). However, urate is an organic anion and could hardly pass through cell membranes in the absence of its transporters, a question remains to be answered: how is extracellular urate transported into cells to exert its effects? Up to now, the related urate transporters include glucose transporter type 9 (Glut9), urate anion transporter 1 (URAT1), organic anion transporter (OAT), ATP-binding cassette subfamily G member 2 (ABCG2) and multi-drug resistance-associated protein 4 (MRP4; Reginato et al., 2012). Among these, Glut9, encoded by solute carrier family 2 member 9 (SLC2A9), is a candidate that could determine the serum urate concentration (Dehghan et al., 2008; Wei et al., 2011). Meanwhile, Glut9 is associated with faster clinical progression and earlier age at onset of PD, whose single nucleotide polymorphisms related to lower urate levels could modify susceptibility to PD (Facheris et al., 2011; González-Aramburu et al., 2013; Simon et al., 2014).

In the present study, we aimed to explore the urate transport mechanism involved in its neuroprotective effects on dopaminergic neurons using MES23.5 dopaminergic cells and urate oxidase (UOx) knockout (KO) mice. Mutation in UOx, a liver-specific uricase that oxidizes urate to allantoin in most mammals, could cause higher levels of urate in blood and cerebrospinal fluid (CSF) in humans (Wu et al., 1992; Paganoni and Schwarzschild, 2017). These findings might provide a novel potential mechanism and direct evidence for investigating antioxidant, such as urate, as a therapeutic target for PD.

## Materials and Methods

### Cell Culture and Treatment

The rodent MES23.5 cell line was a kind gift from Dr. Wei-dong Le at Dalian Medical University (Dalian, China). MES23.5 cells were cultured in Dulbecco’s modified Eagle’s medium (DMEM)-F12 (Gibco, USA) containing Sato’s components growth medium supplemented with 5% fetal bovine serum, 100 units/mL of penicillin and 100 units/mL of streptomycin in a humidified atmosphere containing 5% CO_2_ at 37°C.

For experiments, cells were seeded at a density of 1 × 10^5^/cm^2^ in plates and grown to 70%–80% confluency. MES23.5 cells were pretreated with 100 μM urate (Sigma, Ronkonkoma, NY, USA) for 30 min, and then co-incubated with 50 μM 1-methyl-4-phenylpyridinium (MPP^+^; Sigma, Ronkonkoma, NY, USA) for another 24 h. To investigate urate transport mechanism, MES23.5 cells were pretreated for 30 min with 500 μM uricosuric probenecid (Sigma, Ronkonkoma, NY, USA) to inhibit Glut9-mediated urate transport capacity or 20 μM pifithrin-α (Sigma, Ronkonkoma, NY, USA) to inhibit p53 transcriptional activity prior to the addition of 100 μM urate followed by co-incubation with 50 μM MPP^+^ for 24 h.

### Cell Viability Measurement

Cell viability was measured using 3-(4,5-dimethyl-2-thiazolyl)-2,5-diphenyl-2-H-tetrazolium bromide (MTT) assay. At the end of treatment, culture medium was replaced with the medium containing MTT at a final concentration of 5 mg/mL for 4 h at 37°C. The insoluble formazan was then dissolved in dimethyl sulphoxide (DMSO). Cell viability was assessed at the wavelength of 494 nm and 630 nm using a microplate reader (Molecular Device, M5, Sunnyvale, CA, USA).

### Flow Cytometric Measurement of Reactive Oxygen Species (ROS) and Mitochondrial Transmembrane Potential (Δψm)

The dye 2′,7′-dichlorofluorescein diacetate (H_2_DCFDA) can penetrate into cells, whose oxidation to form the highly fluorescent 2′,7′-dichlorofluorescein (DCF) is proportional to ROS generation. Rhodamine123 (Rh123) can be accumulated into mitochondria via facilitated diffusion. The uptake of Rh123 is decreased due to the reduction of Δψm, which can serve as an indicator of Δψm.

Flow cytometry (Becton Dickinson, USA) was used to measure the changes of ROS and Δψm. At the end of treatment, cells were washed with 2-[4-(2-Hydroxyethyl)-1-piperazinyl] ethanesulfonic acid (HEPES)-buffered saline (HBS) for three times followed by incubation with 5 μM H_2_DCFDA (Molecular Probes, Eugene, OR, USA) or 5 μM Rh123 (Sigma, Ronkonkoma, NY, USA) for 30 min at 37°C in dark. After washing three times with HBS, labeled cells were resuspended in 1 mL HBS. For analysis, 488 nm excitation and 525 nm emission wavelengths were used to assess 10,000 cells for each group. The results were presented as Fluorescence 1-Histogram (FL1-H), setting the gated regions M1 and M2 as markers to observe the fluorescence intensity using CellQuest Software (Becton Dickinson, USA). Fluorescence values of the control were normalized to 100%. The results were expressed as the percentage of fluorescence intensity for each experimental group relative to the control.

### Animals and Treatment

Male 10 month-old UOx KO mice and wild type (WT) littermates mice were used in the present study. The generation of UOx KO mice using a high-performance TALEN strategy was previously described (Lu et al., 2018). Animals were maintained at constant temperature and humidity on a 12-h light/dark cycle with free access to food and water. Mice were intraperitoneal injected with 1-methyl-4-phenyl-1,2,3,6-tetrahydropyridine (MPTP; Sigma, Ronkonkoma, NY, USA) at the dose of 30 mg/kg, or its vehicle saline solution once per day for five consecutive days. Twenty-four hours after the last injection of MPTP, blood and brains were collected for the following studies. One side of the SN was isolated to assess tyrosine hydroxylase (TH) protein levels and striatum was isolated for urate measurement. The other side of the brain was fixed in 4% paraformaldehyde (PFA) for TH immunofluorescent staining. This study was carried out in accordance with the recommendations of National Institutes of Health Guidelines for the Care and Use of Laboratory Animals. The protocol was approved by the Animal Ethics Committee of Qingdao University.

### Serum, Striatal and Intracellular Urate Measurement

For serum urate measurement, mice blood was collected via inner canthus into an anticoagulant tube and then incubated at room temperature for 1 h. After centrifugation at 1500 *g* for 10 min, serum was transferred into Eppendorf tubes and stored at −80°C for urate measurement.

The dissection of striatum was performed as previously described (Glowinski and Iversen, 1966). Briefly, after the removal of rhombencephalon from the brain, a transverse section was made at the level of optic chiasma which separated the cerebrum into two parts. The striatum was dissected with the external walls of the lateral ventricles as medial limits and the corpus callosum as lateral limits. Striatum tissues were weighed and homogenized in 200 μL assay buffer. Then, the homogenate was spinned down at 12,000 *g* for 15 min at 4°C. The supernatant was collected for the striatal urate measurement.

To assess intracellular urate levels, at the end of treatment, MES23.5 cells were washed twice with phosphate-buffered saline (PBS) and harvested. Then, cells were lysed with 200 μL assay buffer and centrifuged at 12,000 *g* for 15 min at 4°C. The resulting supernatant was transferred into Eppendorf tubes for intracellular urate measurement. The protein concentration was determined using the BCA protein assay kit (Thermo Fisher Scientific, USA).

Serum, striatal and intracellular urate levels were assessed using the Uric Acid Fluorometric Assay Kit (BioVision, Milpitas, CA, USA) according to the manufacturer’s instructions. In brief, 20 μL samples were mixed with 30 μL assay buffer in a 96-well plate, followed by adding 50 μL reaction mixture containing 46 μL assay buffer, 2 μL probe and 2 μL enzyme mix. The mixtures were then incubated at 37°C for 30 min in dark. Fluorescence was measured at 535 nm excitation and 590 nm emission wavelengths using a microplate reader. Urate concentration was determined with a standard curve obtained from the defined concentrations of urate. Serum, striatal and intracellular urate concentration were expressed as μM, nmol/mg tissue and μmol/g protein, respectively.

### Immunofluorescent Staining

The brain tissues were sliced into 20 μm-thick sections for TH immunofluorescent staining. After blocking with 10% goat serum for 30 min, the sections were incubated with anti-TH antibody (1:1000, Sigma, Ronkonkoma, NY, USA) overnight at 4°C. Then, sections were incubated with Alexa Fluor^®^ 555 donkey anti-rabbit IgG (H + L) secondary antibody (1:500, Invitrogen, Carlsbad, CA, USA) for 1 h at room temperature, and images were obtained by immunofluorescent microscopy (Observer A1, Zeiss, Germany). The dopaminergic neurons in the SN were outlined on the basis of TH immunofluorescent staining. The number of TH^+^ neurons in the SN was determined using stereological quantification as previously described (Zhang et al., 2015).

### Western Blot

Samples from animals and cells were digested with RIPA lysis buffer (50 mM Tris-HCl, 150 mM NaCl, 1% Nonidet-40, 0.5% sodium deoxycholate, 1 mM EDTA, 1 mM PMSF) and protease inhibitors (Roche Diagnostics, Germany) for 30 min. The lysate was centrifuged at 12,000 *g* for 20 min at 4°C, and the supernatant was used for analysis. Protein concentration was established using the BCA protein assay kit (Thermo Fisher Scientific, USA). A total of 25 μg of protein was electrophoresed and transferred to PVDF membranes. After blocking with 10% non-fat milk for 2 h at room temperature, the membranes were incubated with anti-TH antibody (1:1000, Sigma, Ronkonkoma, NY, USA), anti-superoxide dismutase 1 (SOD1) antibody (1:1000, Santa Cruz Biotechnology, Dallas, TX, USA), anti-Glut9 antibody (1:1000, Abcam, UK) and anti-p53 antibody (1:1000, Cell Signaling Technology, Danvers, MA, USA) overnight at 4°C. Membranes were incubated with horseradish peroxidase-conjugated secondary antibodies (1:10,000, Santa Cruz Biotechnology, Dallas, TX, USA) for 1 h at room temperature. Blots were visualized with UVP Image System and quantified with ImageJ Software.

### Statistical Analysis

SPSS 17.0 was used to analyze the data. One-way analysis of variance (ANOVA) followed by the Student-Newman-Keuls test was used to compare difference between means in more than two groups. Data were presented as mean ± SEM. A probability of *P* < 0.05 was taken to indicate statistical significance.

## Results

### Increased Intracellular Urate Was Responsible for Its Anti-oxidative Effects in MES23.5 Cells

**Figure 1:**
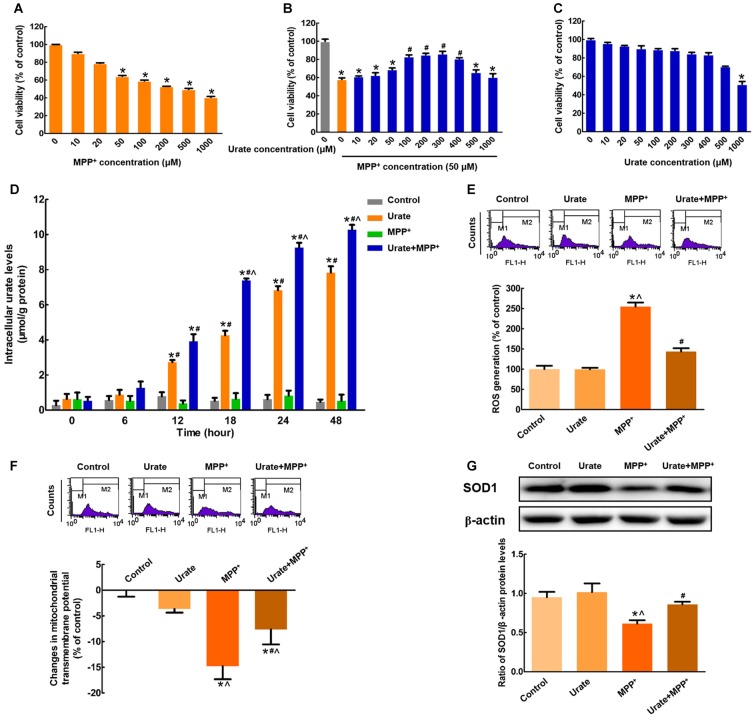
Intracellular urate exerted anti-oxidative effects in MES23.5 cells. **(A)** Dose-dependent toxicity of MPP^+^ in MES23.5 cells. Cell viability was determined by MTT assay. **(B)** Effects of urate on MPP^+^-induced cytotoxicity. Pretreatment with urate antagonized MPP^+^-induced reduction of cell viability at a concentration range of 100–400 μM in MES23.5 cells. **(C)** Effects of urate on cell viability at indicated concentrations. **(D)** Intracellular urate levels were detected using urate fluorometric assay. MPP^+^ treatment induced a significant increase in intracellular urate levels compared with solely urate treatment from 18 h. ROS generation **(E)** and Δψm changes **(F)** of different groups were measured using flow cytometry. **(G)** Western blot was applied to detect SOD1 protein levels. Data were presented as the ratio of SOD1 to β-actin. Urate pretreatment could antagonize MPP^+^-induced excessive ROS generation, collapse of Δψm and decreased SOD1 protein levels. Data were presented as mean ± SEM. *P < 0.05, compared with the control; ^#^*P* < 0.05, compared with the MPP^+^-treated group; ^∧^*P* < 0.05, compared with the urate-treated group, *n* = 6.

In this study, we observed that MPP^+^ treatment for 24 h resulted in a significant decrease in cell viability in a dose-dependent manner in MES23.5 cells (Figure 1A). Considering that 50 μM MPP^+^ reduced cell viability by 35.6% compared with the control, which was the minimum concentration to cause cell damage, 50 μM MPP^+^ was chosen for the following experiments. Pretreatment with indicated concentrations of urate for 30 min could antagonize MPP^+^-induced cytotoxicity at a concentration range of 100–400 μM (Figure 1B). The lowest effective concentration of urate was applied for the following studies. To preclude the possibility that urate caused any toxicity, cells were treated with different doses of urate alone for 24 h. The results showed that urate did not produce any toxic effects on MES23.5 cells except for the highest concentration used (1000 μM; Figure 1C). Therefore, urate pretreatment could significantly antagonize MPP^+^-induced reduction of cell viability in MES23.5 cells.

To elucidate whether exogenous urate could be accumulated into MES23.5 cells, we measured the intracellular urate levels. As shown in Figure 1D, intracellular urate content was gradually increased in a time-dependent manner. Moreover, MPP^+^ treatment induced a 1.7-fold increase of intracellular urate levels compared with solely urate treatment from 18 h. These results suggested that more urate could be accumulated into cells under oxidative stress induced by MPP^+^.

Then, we wanted to illustrate the effects of increased intracellular urate on MPP^+^-induced neurotoxicity. Excessive generation of ROS is a main reason for DNA or RNA damage, which cumulatively contributes to oxidative stress. In the present study, we found that intracellular ROS levels showed a 2.5-fold increase when incubated with 50 μM MPP^+^ for 24 h. While pretreated with 100 μM urate, ROS generation was significantly attenuated (Figure 1E). Moreover, the changes in Δψm were a marker of mitochondrial function, which was also involved in oxidative stress. As shown in Figure 1F, urate pretreatment could remarkably antagonize MPP^+^-induced decrease of Δψm. Next, we measured the protein levels of SOD1, which was a highly potent anti-oxidative agent. It was observed that SOD1 protein levels were decreased by 35.7% in cells treated with MPP^+^, and this effect could be partially reversed by urate pretreatment (Figure 1G). The above results indicated that increased intracellular urate could antagonize MPP^+^-induced neurotoxicity by its anti-oxidative properties.

### Up-regulated Glut9 Accounted for the Increased Intracellular Urate Levels

**Figure 2:**
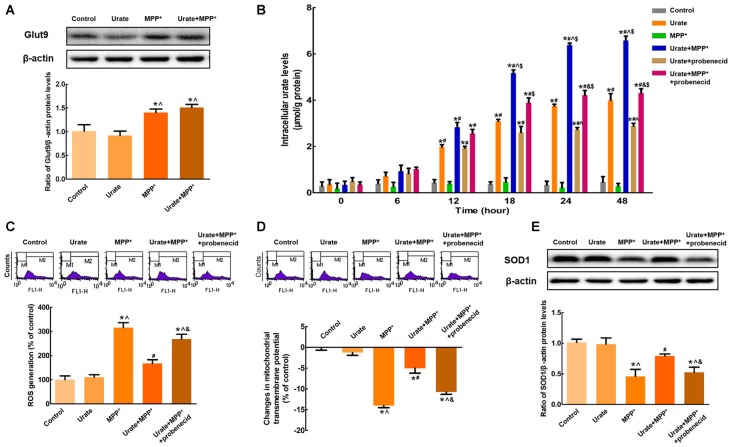
Increased intracellular urate levels were mediated by Glut9.** (A)** Western blot was applied to detect Glut9 protein levels. Data were presented as the ratio of Glut9 to β-actin. Glut9 protein levels were remarkably up-regulated in MPP^+^-treated MES23.5 cells. **(B)** Intracellular urate levels were detected using urate fluorometric assay. After probenecid treatment, intracellular urate levels were obviously decreased. ROS generation **(C)** and Δψm changes **(D)** of different groups were measured using flow cytometry. **(E)** Western blot was applied to detect SOD1 protein levels. Data were presented as the ratio of SOD1 to β-actin. Pretreatment with probenecid completely blocked the anti-oxidative effects of urate in MES23.5 cells. Data were presented as mean ± SEM. *P < 0.05, compared with the control; ^#^*P* < 0.05, compared with the MPP^+^-treated group; ^∧^*P* < 0.05, compared with the urate-treated group; ^&^*P* < 0.05, compared with the urate + MPP^+^-treated group; ^$^*P* < 0.05, compared with the urate + probenecid-treated group, *n* = 6.

Next question is how urate was transported into the cells to exert its anti-oxidative effects. Since the accumulation of urate was transporter-mediated, the expression of high-capacity urate transporter Glut9 was detected in MES23.5 cells. As shown in Figure 2A, Glut9 protein levels were significantly increased by 48.9% when exposed to MPP^+^ compared with the control, indicating an increased cellular ability to transport urate. Then, we specifically inhibited Glut9-mediated urate transport with probenecid, which was also the widely used gout drug. As expected, probenecid could significantly diminish urate uptake from 24 h and intracellular urate levels showed a 1.5-fold decrease with probenecid treatment when compared with urate + MPP^+^ group (Figure 2B). Moreover, we detected the effects of urate on MPP^+^-induced neurotoxicity in the presence of probenecid. Pretreatment of cells with probenecid completely blocked the anti-oxidative effects of urate, resulting in increased ROS generation (Figure 2C), decreased Δψm (Figure 2D) and SOD1 protein levels (Figure 2E). Together, these results indicated that increased intracellular urate levels involved in its neuroprotection were mediated by the up-regulated Glut9.

### p53 Was Responsible for the Up-regulation of Glut9

**Figure 3:**
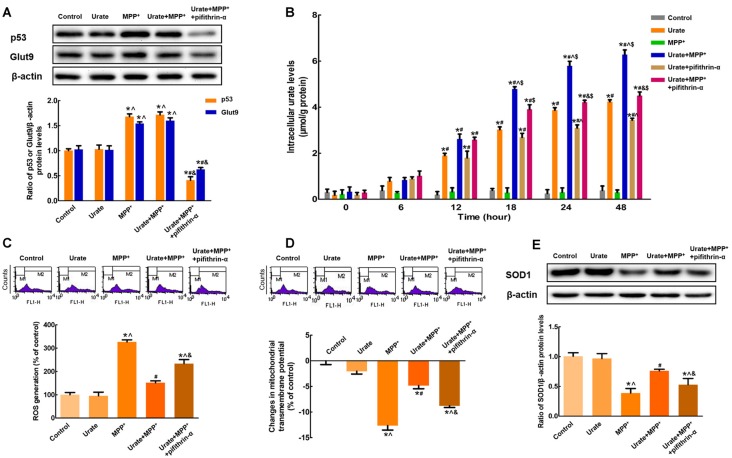
p53 was responsible for the up-regulation of Glut9 in MPP^+^-treated MES 23.5 cells.** (A)** Western blot was applied to detect p53 and Glut9 protein levels. Data were presented as the ratio of p53 or Glut9 to β-actin. The activation of p53 induced an increased Glut9 protein levels in MPP^+^-treated MES23.5 cells. Pretreatment with pifithrin-α reversed the up-regulation of Glut9. **(B)** Intracellular urate levels were detected using urate fluorometric assay. After pifithrin-α treatment, intracellular urate levels were decreased. ROS generation **(C)** and Δψm changes **(D)** of different groups were measured using flow cytometry after the inhibition of p53. **(E)** Western blot was applied to detect SOD1 protein levels. Data were presented as the ratio of SOD1 to β-actin. Pretreatment with pifithrin-α eliminated the anti-oxidative effects of urate in MES23.5 cells. Data were presented as mean ± SEM. *P < 0.05, compared with the control; ^#^*P* < 0.05, compared with the MPP^+^-treated group; ^∧^*P* < 0.05, compared with the urate-treated group; ^&^*P* < 0.05, compared with the urate + MPP^+^-treated group; ^$^*P* < 0.05, compared with the urate + pifithrin-α-treated group, *n* = 6.

The above results suggested that urate could be accumulated into the cells in the presence of urate transporter Glut9. Next, we attempted to explore the possible molecular mechanisms involved in the increased Glut9 expression under oxidative stress. Notably, Glut9 was previously reported to be a direct transcriptional target of tumor suppressor p53 (Itahana et al., 2015). In the present study, we found that p53 protein levels were remarkably increased by 68.3% in MPP^+^-treated MES23.5 cells, which in turn increased the expression of Glut9 (Figure 3A). When incubated with pifithrin-α, which could inhibit the transcriptional activity of p53, the increased expression of Glut9 was found to be abolished. These results indicated that the activation of p53 was responsible for the up-regulation of Glut9 triggered by MPP^+^. Furthermore, under MPP^+^-induced oxidative stress conditions, there was a 1.4-fold decrease in intracellular urate levels with pifithrin-α treatment from 24 h (Figure 3B). Pretreatment with pifithrin-α could also eliminate the anti-oxidative effects of urate against MPP^+^ in MES23.5 cells (Figures 3C–E).

### Up-regulated Nigral Glut9 Levels in UOx KO Mice Antagonized MPTP-Induced Neurotoxicity

**Figure 4:**
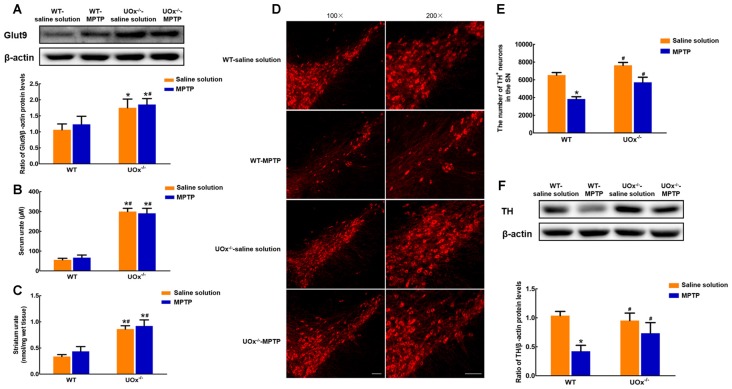
Up-regulated nigral Glut9 alleviated MPTP-induced neurotoxicity in UOx KO mice.** (A)** Western blot was applied to detect Glut9 protein levels. Data were presented as the ratio of Glut9 to β-actin. The nigral Glut9 protein levels were significantly increased in UOx KO mice compared with the WT group. Urate levels were detected using urate fluorometric assay. Urate levels in serum **(B)** and striatum **(C)** were significantly increased in UOx KO mice compared with the WT group. **(D)** Representative pictures showing TH^+^ neurons in the substantia nigra (SN) of different groups determined by immunofluorescent staining. Scale bar = 100 μm. **(E)** Group data showing the number of TH^+^ neurons of different groups. **(F)** Western blot was applied to detect TH protein levels. Data were presented as the ratio of TH to β-actin. The loss of TH^+^ neurons and decreased TH protein levels induced by MPTP were alleviated in UOx KO mice compared with those in WT mice. Data were presented as mean ± SEM. *P < 0.05, compared with the WT-saline solution group; ^#^*P* < 0.05, compared with the WT-MPTP group, *n* = 3.

In UOx KO mice, we found that nigral Glut9 protein levels were significantly increased by 64.6% compared with those of the WT group (Figure 4A). Moreover, urate levels showed a 5.4-fold increase in serum (Figure 4B) and 2.6-fold increase in the striatum (Figure 4C) when compared with those in WT littermates, whose levels were much lower in the brain than that of the blood.

In MPTP-treated UOx KO mice, the reduction in the number of TH^+^ neurons was 25.1% compared with the saline solution-treated group. However, in WT mice, MPTP induced a 41.3% loss of TH^+^ neurons compared with saline solution treatment (Figures 4D,E). Similar results were observed for the nigral TH protein levels. In UOx KO mice, there was a 22.9% reduction in the TH levels after MPTP injection compared with saline solution treatment, while a 59.3% reduction in the WT-MPTP group compared with the WT-saline solution group (Figure 4F). The loss of TH^+^ neurons and decreased TH protein levels induced by MPTP were remarkably reversed in UOx KO mice compared with those in WT littermates.

## Discussion

In the present study, we demonstrated that Glut9-mediated increased urate uptake was involved in the protection of dopaminergic cells against MPTP/MPP^+^-induced neurotoxicity both *in vivo* and* in vitro*.

Urate, primarily as a weak acid, is generated from purine metabolism (So and Thorens, 2010). In the majority of mammals, urate undergoes oxidative degradation by UOx to form the more soluble compound allantoin. By contrast, in humans and primates, urate constitutes the end product of purine metabolism due to the absence of functional UOx. Thus, urate levels in humans are approximately 10 times more than those in most mammals, which has been hypothesized to reflect an evolutionary advantage and lengthening lifespan against aging (Mandal and Mount, 2015). Previous studies have reported that urate levels in postmortem brains of PD patients are lower compared with the control (McFarland et al., 2013). Numerous epidemiological studies have also shown serum or CSF urate levels were negatively correlated with the risk and severity of PD (de Lau et al., 2005; McFarland et al., 2013; Gao et al., 2016; Andersen et al., 2017).

In the present study, we observed a direct protection by urate against MPTP/MPP^+^-induced neurotoxicity both *in vivo* and *in vitro*. MPTP, as a common agent for generating PD animal models (Jiang et al., 2017), is first metabolized by monoamine oxidase-B (MAO-B) to 1-methyl-4-phenyl-2,3-dihydropyridinium (MPDP^+^), and then deprotonates to generate MPP^+^, which can enter cells through the dopamine reuptake system and inhibit complex I of the mitochondrial respiratory chain to induce oxidative stress (Desai et al., 1996; Cassarino et al., 1999; Smeyne and Jackson-Lewis, 2005; Shen et al., 2017). Previous studies have reported that there exists xanthine oxidase enzyme in brain converting xanthine to urate, so that the brain has the capacity to generate urate *in situ*. Furthermore, urate generated peripherally could also access to the brain (Bowman et al., 2010). In our study, using UOx KO mice, which have been reported as a suitable model of hyperuricemia and more closely mimic purine metabolism in humans (Lu et al., 2018), we provided the evidence that serum and striatal urate levels were significantly elevated. Specific localization of urate transporters in brain raised the possibility that urate might be partly transported into ventricular CSF. CSF-derived urate could also increase the regional urate levels near the ventricle, for example in the striatum (Tomioka et al., 2016). In addition, MPTP could induce an increased trend of striatal urate levels, indicating that urate might be induced to alleviate the neurotoxicity triggered by MPTP. Moreover, we found that elevated urate levels could alleviate the loss of nigral dopaminergic neurons and protect MES23.5 cells against MPP^+^-induced cytotoxicity by its anti-oxidative properties. These findings are consistent with some previous studies. Urate treatment could attenuate the impairment of motor performance in 6-hydroxydopamine (6-OHDA)-lesioned rat models of PD (Gong et al., 2012). *In vitro*, urate could block cell injury induced by 6-OHDA, dopamine and rotenone in dopaminergic neurons (Jones et al., 2000; Duan et al., 2002; Zhu et al., 2012). Lowering serum and striatal urate levels by oral allopurinol potentiate striatal dopamine loss but could not exacerbate dopaminergic neuron degeneration in a dual pesticide mice model of PD (Kachroo and Schwarzschild, 2014).

Although the beneficial effects of urate have been demonstrated, the underlying molecular mechanism is still largely unknown. Urate, in fact, is a natural antioxidant and a powerful scavenger of free radicals, which accounts for more than two-thirds of the antioxidant capacity in humans (Ames et al., 1981). Recently, several studies on the mechanisms for neuroprotection by urate have focused on its anti-oxidative properties. It has been reported that urate could induce nuclear factor E2 related factor 2 (Nrf2) nuclear translocation and further regulated the expression of antioxidant response elements (ARE; Zhang et al., 2014), which in turn remarkably increase the synthesis and release of glutathione (GSH; Bakshi et al., 2015). Furthermore, protein kinase B (Akt)/glycogen synthase kinase 3β (GSK3β) pathway might be involved in the beneficial effects of urate against the toxicity of 6-OHDA (Gong et al., 2012). It should be noted that the anti-oxidative ability of urate might be dependent on its intracellular levels. Although one study reported that urate could not be accumulated into rat mesencephalic dopaminergic neurons, indicating the protective effects of urate occurred probably extracellularly (Guerreiro et al., 2009), most studies consistently agreed that exogenous urate could increase intracellular urate levels through some unknown transport mechanisms (Cipriani et al., 2012a,b; Zhang et al., 2014). This discrepancy might in part be explained by different urate measurement protocols. Thus, in the present study, we attempted to investigate the urate transport mechanism responsible for its neuroprotective effects under oxidative stress conditions.

Urate transporters, such as Glut9, URAT1, ABCG2 and OAT, play a key role in maintaining urate homeostasis. Glut9, encoded by *SLC2A9* gene, is a newly identified high-capacity urate transporter (Caulfield et al., 2008; Vitart et al., 2008; Preitner et al., 2009). It is widely expressed in liver, lung, heart, kidney, intestine, leucocytes and chondrocytes. Recent studies show that Glut9 is also observed in neurons and brain capillaries (Tomioka et al., 2016). Glut9-mediated urate transport is probably not a coupled transport system but rather a urate uniporter (Bibert et al., 2009). Genome-wide association studies (GWAS) has identified variation in *SLC2A9* gene is the strongest known genetic determinant of plasma urate concentration in humans (Dehghan et al., 2008; Köttgen et al., 2013). An interaction between genome-wide variant and serum urate levels may be a predictor of disease progression and important step for personalizing prognosis in PD (Nazeri et al., 2015). In this study, we found that the expression of Glut9 was up-regulated with MPP^+^ treatment, resulting in an increased capacity for urate uptake.

Notably, recent studies have reported that *SLC2A9* is a direct transcriptional target of p53 (Itahana et al., 2015), which further regulates multiple antioxidant genes, implying a substantial anti-oxidative function of p53-dependent pathway in the protection of cells (Tan et al., 1999; Bensaad et al., 2006; Cano et al., 2009; Hu et al., 2010; Zhou et al., 2013). A growing number of signals can activate p53, including DNA damage, oncogene activation, nitrative and oxidative stress, hypoxia and more (Horn and Vousden, 2007). In the present study, we found that the expression of p53 was remarkably increased when exposed to MPP^+^, and further induced the increased Glut9 protein levels. Therefore, the activation of p53 played a causal role in the up-regulation of Glut9. As a result, the transport of urate into cells mediated by Glut9 was increased. After the application of probenecid or pifithrin-α, intracellular urate levels were significantly decreased and its neuroprotective effects were also found to be abolished. The ability to adapt to oxidative stress is an important component of cellular defense strategy in mammalian cells (Wiese et al., 1995). In this study, the relatively high intracellular urate levels may serve as an endogenous compensatory mechanism for resistance to oxidative stress. These adaptive effects are accompanied by, and may be consequences of defects in cellular anti-oxidative stress system.

**Figure 5:**
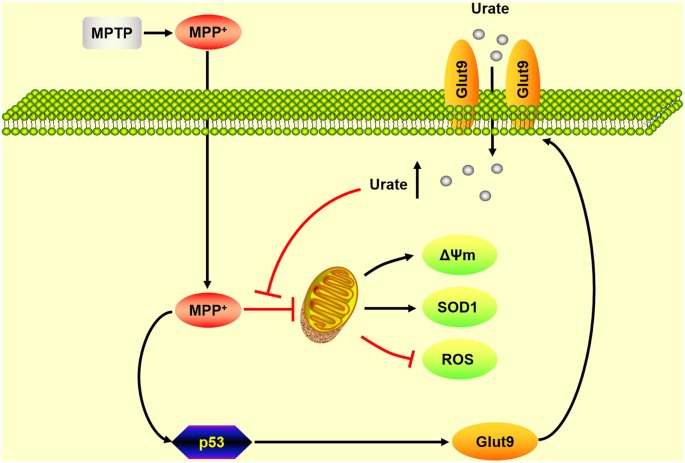
A schematic diagram of the mechanism underlying the neuroprotective effects of urate on dopaminergic neurons. Under oxidative stress induced by MPP^+^, the activation of p53 induced an increased expression of its transcriptional target Glut9. Increased urate uptake mediated by Glut9 could protect dopaminergic neurons against MPP^+^-induced neurotoxicity by its anti-oxidative properties.

As summarized in Figure 5, our present findings suggest that up-regulation of Glut9, which was caused by the activation of p53, was responsible for the increased urate uptake in the MPP^+^-induced cell model and the MPTP-induced mice model of PD. Increased intracellular urate antagonized MPP^+^-induced excessive ROS generation, collapse of Δψm and decreased SOD1 levels to exert its anti-oxidative effects. Our study demonstrated that Glut9-mediated urate uptake was essential for its neuroprotective effects which strengthened the rationale for exploring urate-elevating strategies as potential therapeutic targets for PD. Nevertheless, what is considered a “normal range” without increasing systemic urate levels to avoid associated side effects should be further investigated.

## Author Contributions

HJ and MB conceived the project and designed the study. MB, QJ and XD performed the experiments, analyzed data and interpreted results. MB wrote the manuscript. HJ and QJ reviewed and edited the manuscript. All authors have read and approved the final version of the manuscript.

## Conflict of Interest Statement

The authors declare that the research was conducted in the absence of any commercial or financial relationships that could be construed as a potential conflict of interest.

## Abbreviations

ABCG2: ATP-binding cassette subfamily G member 2
Akt: protein kinase B
ARE: antioxidant response elements
CSF: cerebrospinal fluid
Glut9: glucose transporter type 9
GSK3β: glycogen synthase kinase 3β
MAO-B: monoamine oxidase-B
MPDP^+^: 1-methyl-4-phenyl-2,3-dihydropyridinium
MPP^+^: 1-methyl-4-phenylpyridinium
MPTP: 1-methyl-4-phenyl-1,2,3,6-tetrahydropyridine
MRP4: multi-drug resistance-associated protein 4
MTT: 3-(4,5-dimethyl-2-thiazolyl)-2,5-diphenyl-2-H-tetrazolium bromide
Nrf2: nuclear factor E2 related factor 2
OAT: organic anion transporter
PD: Parkinson’s disease
ROS: reactive oxygen species
SLC2A9: solute carrier family 2 member 9
SN: substantia nigra
SOD1: superoxide dismutase 1
TH: tyrosine hydroxylase
UOx: urate oxidase
URAT1: urate anion transporter 1
6-OHDA: 6-hydroxydopamine
Δψm: mitochondrial transmembrane potential.
